# PARP-1 promotes autophagy via the AMPK/mTOR pathway in CNE-2 human nasopharyngeal carcinoma cells following ionizing radiation, while inhibition of autophagy contributes to the radiation sensitization of CNE-2 cells

**DOI:** 10.3892/mmr.2015.3604

**Published:** 2015-04-09

**Authors:** ZE-TAN CHEN, WEI ZHAO, SONG QU, LING LI, XIAO-DI LU, FANG SU, ZHONG-GUO LIANG, SI-YAN GUO, XIAO-DONG ZHU

**Affiliations:** 1 Department of Radiation Oncology, Affiliated Cancer Hospital of Guangxi Medical University, Cancer Institute of Guangxi Zhuang Autonomous Region, Nanning, Guangxi 530021, P.R. China; 2 Key Laboratory of High-Incidence-Tumor Prevention and Treatment, Guangxi Medical University, Ministry of Education, Nanning, Guangxi 530021, P.R. China

**Keywords:** autophagy, nasopharyngeal carcinoma, poly-(adenosine diphosphate-ribose) polymerase-1, adenosine monophosphate-activated protein kinase, mammalian target of rapamycin

## Abstract

It was previously reported that poly-(adenosine diphosphate-ribose) polymerase-1 (PARP-1) regulated ionizing radiation (IR)-induced autophagy in CNE-2 human nasopharyngeal carcinoma cells. The present study aimed to investigate whether PARP-1-mediated IR-induced autophagy occurred via activation of the liver kinase B1 (LKB1)/adenosine monophosphate-activated protein kinase (AMPK)/mammalian target of rapamycin (mTOR) signaling pathway in CNE-2 cells. In addition, the effect of PARP-1 and AMPK inhibition on the radiation sensitization of CNE-2 cells was investigated. CNE-2 cells were treated with 10 Gy IR in the presence or absence of the AMPK activator 5-amino-1-β-D-ribofuranosyl-1H-imidazole-4-carboxamide (AICAR). In addition, IR-treated CNE-2 cells were transfected with lentivirus-delivered small-hairpin RNA or treated with the AMPK inhibitor Compound C. Western blot analysis was used to assess the protein expression of PARP-1, phosphorylated (p)-AMPK, microtubule-associated protein 1 light chain 3 (LC3)-II and p-P70S6K. Cell viability and clone formation assays were performed to determine the effect of *PARP-1* silencing and AMPK inhibition on the radiation sensitization of CNE-2 cells. The results showed that IR promoted PARP-1, p-AMPK and LC3-II protein expression as well as decreased p-P70S6K expression compared with that of the untreated cells. In addition, AICAR increased the expression of p-AMPK and LC3-II as well as decreased p-P70S6K expression compared with that of the IR-only group; however, AICAR did not increase PARP-1 expression. Furthermore, *PARP-1* gene silencing decreased the expression of PARP-1, p-AMPK and LC3-II as well as increased p-P70S6K expression. Compound C decreased p-AMPK and LC3-II expression as well as increased p-P70S6K expression; however, Compound C did not increase PARP-1 expression. Western blot analysis detected limited expression of p-LKB1 in all treatment groups. Cell viability and clone formation assays revealed that PARP-1 or AMPK inhibition reduced the proliferation of CNE-2 cells following IR. In conclusion, the present study demonstrated that PARP-1 promoted autophagy via the AMPK/mTOR pathway; in addition, PARP-1 or AMPK inhibition contributed to the radiation sensitization of CNE-2 cells following IR. However, it remains to be elucidated whether PARP-1 is an upstream mediator of the LKB1 pathway in CNE-2 cells following IR.

## Introduction

Nasopharyngeal carcinoma (NPC) is a type of malignant neoplasm, which is prevalent in Southeast Asia and Southern China, with incidence rates of 20–30 per 100,000 males and 15–20 per 100,000 females. In addition, NPC accounts for ~50,000 mortalities annually worldwide and ~80,000 novel cases are diagnosed each year (1,2). NPC is a radiosensitive neoplasm, for which radiotherapy (RT) is the primary treatment option. RT treatment of early stage NPC is effective in 85% of patients; however, for loco-regional advanced NPC, current treatments are ineffective (3). Radioresistance remains to be an obstacle for the successful treatment of NPC in numerous cases (4,5) and accounts for the majority of treatment failures, resulting in relapse and metastasis in NPC patients following RT (6).

Autophagy involves the degradation of long-lived proteins and cytoplasmic organelles, the products of which are recycled for the production of macromolecules and adenosine triphosphate (ATP) in order to maintain cellular homeostasis (7). Therefore, autophagy is an important survival mechanism in response to several types of stresses, including nutrient starvation, hypoxia, overcrowding, high temperatures as well as the accumulation of damaged or expendable organelles and cytoplasmic components (7,8). Microtubule-associated protein 1 light chain 3 (LC3)-II is a marker of autophagy (9); increased LC3-II levels indicate that the autophagy has been initiated. During autophagic degradation, LC3-II is converted back to LC3-I through protease cleavage (10). Previous studies have demonstrated that autophagy is initiated in various types of cancer cells in response to anticancer therapies (11–17). Autophagy was reported to be induced by ionizing radiation (IR) in certain types of cancer cells, including malignant glioma cells (11–13). In addition, genetic knockout studies of autophagy-associated genes were demonstrated to enhance the development of spontaneous malignancies, whereas mice deficient in autophagy-associated genes showed increased sensitivity to radiotherapy (14–17). Therefore, elucidating the mechanisms involved in autophagy may provide effective strategies for improving the radiation sensitization of NPC.

Poly [adenosine diphosphate (ADP)-ribose] polymerase-1 (PARP-1) is a nuclear enzyme which binds DNA by two zinc finger motifs and transfers chains of ADP-ribosyl moieties (PARs) from nicotinamide-adenine-dinucleotide (NAD^+^) to chromatin-associated acceptor proteins (18). This post-translational modification has an important role in facilitating DNA repair through releasing PARP-1 from DNA and allowing for the recruitment of proteins involved in both base excisional repair and homologous recombination (18,19). Irradiation is known to induce DNA damage and activate PARP-1 (20). PARP-1 and DNA-dependent protein kinase-deficient cell lines were reported to be 4-fold more sensitive to IR; in addition, these cells demonstrated reduced potentially lethal damage recovery (PLDR) in G_0_ cells compared with that of their proficient counterparts (21). A previous study demonstrated that PARP-1 mediated IR-induced autophagy and PARP-1 inhibition resulted in the radiation sensitization of CNE-2 cells (22). In addition, the liver kinase B1 (LKB1)/adenosine monophosphate (AMP)-activated protein kinase (AMPK)/mammalian target of rapamycin (mTOR) signaling pathway has been reported to link cellular metabolism and energy status to the signal transduction pathways involved in cell growth, proliferation and autophagy (23,24). Mouse embryonic fibroblasts (MEFs) with *Bax*/*Bak* double-knockout (*Bax−*/*−Bak−*/*−*) is a well-established model for studying necrotic cell death; these cells were used to determine a novel regulatory function of PARP-1 in autophagy via the activation of serine/threonine protein kinase LKB1 and AMPK as well as the subsequent suppression of mTOR. These results indicated that autophagy served as a cell survival mechanism to counteract reactive oxygen species-mediated necrosis (25).

The LKB1/AMPK/mTOR signaling pathway was extensively studied in metabolic disorders and evidence has suggested its implication in cancer cell biology (23,24). In addition, PARP-1 was reported to be a crucial for the repair of radiation-induced single-strand DNA breaks (26). PARP inhibitors have demonstrated promising results for use in cancer therapy; however, certain limitations have arisen which require further investigation. The chemical structures of PARP inhibitors are highly varied; however, they have short half-lives and so require frequent administration, which contributes to poor patient compliance (27). In addition, long-term inhibition of PARP activity may result in novel mutations or other unknown adverse effects; therefore, the long-term effects of these drugs require verification (28,29). It was hypothesized that the regulation of autophagy in CNE-2 cells following IR occurred via the PARP-1/LKB1/AMPK/mTOR pathway. Thus, the elucidation of this mechanism may provide evidence for the use of PARP-1, LKB1 or AMPK inhibitors and mTOR activators as adjuvant therapies for the treatment of NPC. The present study aimed to investigate whether the mechanism of PARP-1-mediated IR-induced autophagy in CNE-2 cells proceeded via activation of the LKB1/AMPK/mTOR signaling pathway. In addition, the effect of PARP-1 and AMPK inhibition on the radiation sensitization of CNE-2 cells was investigated. Pharmacological or genetic regulation of these pathways may be a potential strategy to enhance radiosensitivity of NPC.

## Materials and methods

### Cell culture

CNE-2 human nasopharyngeal carcinoma cells were purchased from the Cancer Hospital of Shanghai Fudan University (Shanghai, China) (30). Cells were cultured in RPMI-1640 medium (HyClone Laboratories, Inc., Logan, UT, USA) supplemented with 10% fetal bovine serum (Gibco-BRL, Carlsbad, CA, USA), penicillin (100 U/ml), streptomycin (100 U/ml) (North China Pharmaceutical Group Corp., Shijiazhuang, China) and were maintained in a humidified 5% CO_2_ atmosphere at 37°C.

### Reagents

5-amino-1-β-D-ribofuranosyl-1H-imidazole-4-car boxamide (AICAR), an activator of AMPK, was purchased from Cayman Chemical (Ann Arbor, MI, USA). 6-[4-(2-Piperidin-1 -yl-ethoxy)-phenyl)]-3-pyridin-4-yl-pyrrazolo [1,5-a]-pyrimidine (Compound C), an inhibitor of AMPK, was purchased from Merck Millipore Calbiochem (Darmstadt, Germany). Rabbit polyclonal anti-PARP-1 (1:1,000; cat. no. 9542S), rabbit monoclonal anti-phospho-LKB1-Ser428 (p-LKB1; 1:1,000; cat. no. 3482S), rabbit monoclonal anti-phospho-AMPK-Thr172 (p-AMPK; 1:2,000; cat. no. 4188S) and rabbit polyclonal anti-phospho-P70S6K-T421/S424 (p-P70S6K; 1:1,000; cat. no. 9204S) primary antibodies were purchased from Cell Signaling Technology (Danvers, MA, USA). Rabbit polyclonal anti-LC3-II primary antibody (1:1,000; cat. no. L7543) was purchased from Sigma-Aldrich (Shanghai, China). Rabbit polyclonal GAPDH primary antibody (1:5,000; cat. no. 10494-1-AP) was purchased from Proteintech Group, Inc. (Chicago, IL, USA) and the fluorescent-labeled goat anti-rabbit immunoglobulin G (IgG) secondary antibody (1:15,000; cat. no. 7054) was purchased from Cell Signaling Technology. Lysis buffer, a mixture of radioimmunoprecipitation assay and phenyl-methylsulfonyl fluoride, was purchased from Beyotime Institute of Biotechnology (Shanghai, China). A PhosSTOP tablet (Roche, Basel, Switzerland) was also dissolved in lysis buffer. 3-(4, 5-dimethylthiazol-2-yl)-2,5-diphenyltetrazolium bromide (MTT), dimethyl sulfoxide (DMSO), 100% methanol and Giemsa solution were purchased from Solarbio Science and Technology Co., Ltd (Beijing, China).

### Fluorescence microscopy

*PARP-1* gene silencing of CNE-2 cells was established as previously described (31). For the lentiviral infection, CNE-2 cells were cultured in 6-well plates. Subsequently, the PARP-1-shRNA-expressing lenti-virus (Shanghai Genechem Biotechnology, Shanghai, China) was added, with a multiplicity of infection of 20 in the CNE-2 cells for 8 h. The transduction efficiency was determined using an inverted fluorescence microscope (IX71; Olympus Corp., Beijing, China).

### Irradiation of CNE-2 cells

IR was performed using 6-MV X-rays with a linear accelerator (Precise 1120, Elekta Instrument AB, Stockholm, Sweden), at a dose rate of 220 cGy/min (source-to-surface distance, 100 cm).

### Western blot analysis

CNE-2 cells were washed with ice-cold phosphate-buffered saline (PBS) twice and lysed with lysis buffer at 4°C for 30 min. The lysates were then centrifuged at 4°C for 15 min at a centrifugal acceleration of 18,500 x g. Protein content in the supernatants was determined using the Bicinchoninic Acid Protein Assay kit (Beyotime Institute of Biotechnology). In order to detect PARP-1, equal amounts of protein (50 *µ*g) were loaded onto a 7% SDS-polyacrylamide gel, for p -LK B1-Ser428, p-A M PK-T h r172 and p-P70S6K-T421/S424 detection, equal amounts of protein were loaded onto a 10% SDS-polyacrylamide gel. A 15% SDS-polyacrylamide gel was used to detect equal amounts of LC3-II protein. Following electrophoresis, proteins were transferred onto polyvinylidene fluoride membranes (Merck Millipore, Billerica, MA, USA). Membranes were then blocked with milk for 1 h and then were incubated with following primary antibodies at 4°C overnight: Anti-PARP-1 (1:1,000), anti-p-LKB1-Ser428 (1:1,000), anti-p-AMPK-T172 (1:2,000), anti-p-P70S6K-T421/S424 (1:1,000), anti-GAPDH (1:5,000) and anti-LC3-II (1:1,000). Following washing and incubating with fluorescent-labeled goat anti-rabbit IgG secondary antibody (1:15,000) at room temperature for 1 h, the fluorescence intensities of the blots were detected using the Odyssey Infrared Imaging system version 3.0.X (LI-COR Biosciences, Lincoln, NE, USA).

### MTT assay

Cells were prepared at a concentration of 1×10^4^ cells/ml and seeded onto 96-well plates at 100 *µ*l/well. Following 24 h of adherence, the cells were treated with Compound C for 24 h, then incubated for 0, 1, 2, 3 or 4 days. An MTT assay was performed by adding 20 *µ*l MTT (5 mg/ml) to wells and incubating for 3 h in the dark. Subsequently, the supernatants were removed. A total of 150 *µ*l DMSO was added to each well and after 15 min the absorbance value (optical density, OD) of each well was measured using a microplate reader (MK3; Thermo Fisher Scientific, Shanghai, China) at 492 nm. The MTT assay procedure was used to determine the proliferation rate of *PARP-1*-silenced CNE-2 cells as well as control IR-treated and untreated CNE-2 cells. All experiments were performed in triplicate.

### Plate clone formation assay

Cells were seeded at a density of 5×10^3^ cells/well into 6-well plates. Following 24 h of adherence, the cells were treated with Compound C (10 *µ*M) for 24 h, then incubated at 37°C for 10 days. Cells were then washed twice with PBS and fixed in 100% methanol for 30 min, prior to staining with Giemsa solution for 30 min. The number of colonies containing ≥50 cells were counted under a microscope (IX71). The clone formation assay was also applied to *PARP-1*-silenced CNE-2 cells as well as control IR-treated and untreated CNE-2 cells. All experiments were performed in triplicate.

### Statistical analysis

Values are expressed as the mean ± standard deviation. Data were analyzed using the one-way analysis of variance and least significant difference tests with SPSS 16.0 software (SPSS, Inc., Chicago, IL, USA) to determine statistical significance. P<0.05 was considered to indicate a statistically significant difference between values.

## Results

### IR induces autophagy in CNE-2 cells

**Figure 1:**
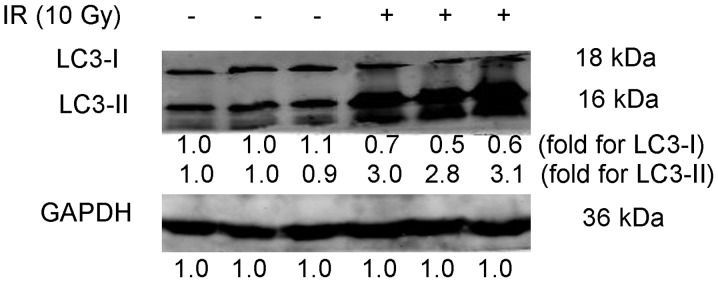
Irradiation induces autophagy in CNE-2 cells. At 48 h post treatment with 10 Gy IR, western blot analysis was used to determine levels of LC3-I and LC3-II in untreated cells and IR-treated cells. GAPDH was used as a loading control. LC3-I and LC3-II levels were quantified and expressed as the mean fold change relative to GAPDH for three independent experiments. IR, ionizing radiation; LC3, microtubule-associated protein 1 light chain 3.

LC3-II is a marker of autophagy, the accumulation of which suggests the process of autophagy; therefore, the autophagic response was measured through the conversion of LC3-I to LC3-II (32). In the present study, protein expression levels of LC3-I and LC3-II in CNE-2 cells treated with 10 Gy IR were compared with those in the untreated control group CNE-2 cells. At 48 h post IR, CNE-2 cells were collected and subjected to western blot analysis. The relative density of LC3-I and LC3-II to GAPDH levels were then determined and the mean fold change of three independent experiments was calculated. As shown in Fig. 1, following IR, the relative density of LC3-I (0.6±0.1) was significantly decreased compared with that of the untreated control group (1.0±0.2; P<0.05). In addition, the relative density of LC3-II in cells treated with IR (3.0±0.2) was significantly increased compared with that of the untreated control group (1.0±0.1; P<0.05). This implied that LC3-I was converted to LC3-II following IR, therefore indicating that IR induced autophagy in CNE-2 cells.

### Expression of PARP-1 in CNE-2 cells

**Figure 2:**
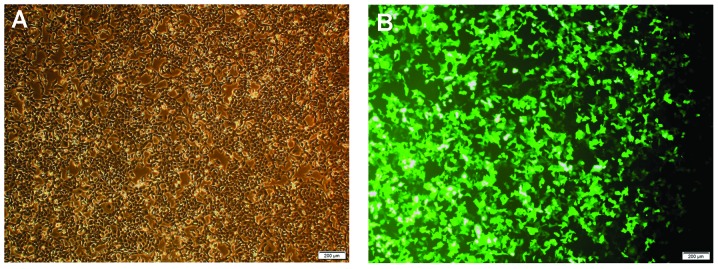
Representative microscopy images of CNE-2 cells following *PARP-1*-silencing by lentivirus-delivered small-hairpin RNA transfection. (A) Phase contrast image of transfected CNE-2 cells. (B) Fluorescence microscopy image of green fluorescent protein expression of cells. Images were captured of cells in an identical field of vision (scale bar, 200 *µ*m). PARP-1, poly-(adenosine diphosphate-ribose) polymerase-1.

**Figure 3:**
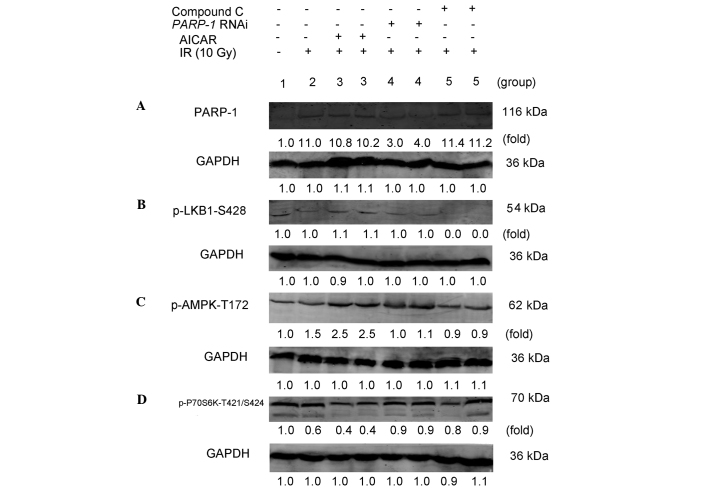
PARP-1 promotes autophagy through the AMPK/mammalian target of rapamycin pathway in CNE-2 cells following IR. CNE-2 cells in each group were treated as follows: 1, untreated control group); 2, 10 Gy IR; 3, pretreatment with AICAR (2.0 mM) for 2 h + 10 Gy IR; 4, *PARP-1* RNAi using lentivirus-delivered small-hairpin RNA transfection + 10 Gy IR; 5, pretreatment with Compound C (10 *µ*M) for 2 h + 10 Gy IR. (A) After 48 h, CNE-2 cells were collected and subjected to western blot analysis for the detection of PARP-1. After 30 min, CNE-2 cells were collected and subjected to western blot for detection of (B) p-LKB1-S428, (C) p-AMPK-Thr172 and (D) p-P70S6K-T421/S424. GAPDH was used as the loading control. Protein expression levels of PARP-1, p-LKB1-Ser428, p-AMPK-Thr172 and p-P70S6K-T421/S424 were then quantified and the mean fold change relative to GAPDH for three independent experiments. PARP-1, poly-(adenosine diphosphate-ribose) polymerase-1; AMPK, adenosine monophosphate-activated protein kinase; IR, ionizing radiation; AICAR, 5-amino-1-β-D-ribofuranosyl-1H-imidazole-4-carboxamide; RNAi, RNA interference; Compound C, 6-[4-(2-Piperidin-1-yl-ethoxy)-phenyl)]-3-pyridin-4-yl-pyrrazolo [1,5-a]-pyrimidine; LKB1, liver kinase B1, p-, phosphorylated.

CNE-2 cells were transfected with LV-shRNA in order to silence the *PARP-1* gene. The lentivirus contained a gene encoding green fluorescent protein; therefore, effectively transfected CNE-2 cells would appear green under an inverted fluorescence microscopy. As shown in the photomicrographs in Fig. 2, the majority of CNE-2 cells were effectively transfected with LV-shRNA. The protein expression levels of PARP-1 were then determined using western blot analysis. As shown in Fig. 3A, the relative density of PARP-1 in IR-treated *PARP-1*-silenced cells (3.5±0.5) was significantly decreased compared with that of the IR-only treatment group (11.0±0.5; P<0.05); this therefore confirmed that LV-shRNA effectively silenced *PARP-1* in CNE-2 cells. In addition, the relative density of PARP-1 in IR-treated cells (11.0±0.5) was markedly increased compared with that of the untreated CNE-2 cells (1.0±0.4; P<0.05), therefore indicating that IR promoted the activation of PARP-1. Furthermore, following IR, CNE-2 cells were treated with an AMPK activator (2.0 mM AICAR) or inhibitor (10 *µ*M Compound C). The results revealed that there were no significant differences in the relative density of PARP-1 expression between the AICAR (10.5±0.3) or Compound C (11.3±0.4) treatment groups and the IR-only treatment group (11.0±0.5; P>0.05). This therefore indicated that the activation or inhibition of AMPK did not effect the expression of PARP-1.

### Expression of p-LKB1-Ser428 in CNE-2 cells

As shown in Fig. 3B, dim bands were observed for p-PKB1-Ser428 in untreated CNE-2 cells, IR-treated CNE-2 cells in the presence and absence of AICAR as well as IR-treated *PARP-1*-silenced cells. These bands were too dim to detect any changes, which indicated that p-LKB1-Ser428 was expressed in CNE-2 cells whether they were treated with IR or not and *PARP-1* gene silencing did not decrease p-LKB1-Ser428. No bands were detected for the IR-treated cells with Compound C, which indicated that Compound C may block the expression of p-LKB1-Ser428 in CNE-2 cells.

### Expression of p-AMPK-Thr172 in CNE-2 cells

As shown in Fig. 3C, following IR, the relative density of p-AMPK-Thr172 in CNE-2 cells (1.5±0.1) was increased compared with that of the untreated cells (1.0±0.1; P<0.05). This indicated that IR promoted the expression of AMPK in CNE-2 cells. The relative density of p-AMPK in AICAR-treated cells following IR (2.5±0.2) was significantly increased compared with that of the IR-only treatment group (1.5±0.1; P<0.05), confirming that AICAR promoted AMPK expression in IR-treated cells. Following *PARP-1* silencing, the relative density of IR-treated cells (1.1±0.1) was markedly decreased compared with that of the IR-only treatment group (1.5±0.1; P<0.05). This indicated that *PARP-1* gene silencing reduced the expression of AMPK following IR. Furthermore, p-AMPK-Thr172 expression in Compound C-treated cells following IR (0.9±0.2) was significantly decreased compared with that of the IR-only treatment (1.5±0.1; P<0.05).

### Expression of p-P70S6K-T421/S424 in CNE-2 cells

mTOR inhibits autophagy predominantly through activating the downstream molecule p70S6K (33). As shown in Fig. 3D, following IR, the relative density of p-p70S6K in CNE-2 cells (0.6±0.2) was decreased compared with that of the untreated cells (1.0±0.1; P<0.05), suggesting that IR attenuated the expression of p-P70S6K in CNE-2 cells. The relative density of p-P70S6K in AICAR-treated cells following IR (0.4±0.1) was significantly decreased compared with that of the IR-only treatment group (0.6±0.2; P<0.05). This indicated AICAR reduced mTOR activity in CNE-2 cells following IR. By contrast, *PARP-1* silencing markedly increased the relative density of p-P70S6K in IR-treated cells (0.9±0.1) compared with that of the IR-only treatment group (0.6±0.2; P<0.05), which suggested that *PARP-1* gene silencing induced mTOR activity following IR. The relative density of p-P70S6K in Compound C-treated cells following IR was significantly increased compared with that of the IR-only treatment group (0.6±0.2; P<0.05), indicating that AMPK inhibition enhanced mTOR activity following IR.

### Expression of LC3-II in CNE-2 cells

**Figure 4:**
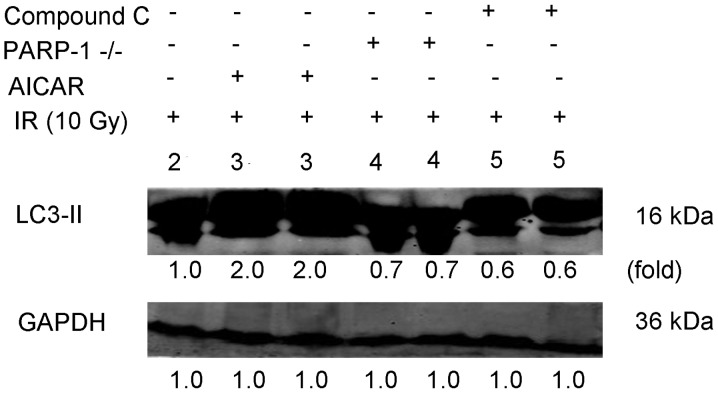
Expression of LC3-II in IR-treated CNE-2 cells. CNE-2 cells in each group were treated as follows: 2, 10 Gy IR; 3, pretreatment with AICAR (2.0 mM) for 2 h + 10 Gy IR; 4, *PARP-1* RNA interference using lentivirus-delivered small-hairpin RNA transfection + 10 Gy IR; 5, pretreatment with Compound C (10 *µ*M) for 2 h + 10 Gy IR. After 48 h, CNE-2 cells were collected and subjected to western blot analysis for detection of LC3-II. GAPDH was used as the loading control. Protein expression levels of LC3-II were then quantified and the mean fold change relative to GAPDH for three independent experiments. LC3, micro-tubule-associated protein 1 light chain 3; IR, ionizing radiation; AICAR, 5-amino-1-β-D-ribofuranosyl-1H-imidazole-4-carboxamide; PARP-1, poly-(adenosine diphosphate-ribose) polymerase-1; Compound C, 6-[4-(2-Piperidin-1-yl-ethoxy)-phenyl)]-3-pyridin-4-yl-pyrrazolo [1,5-a]-pyrimidine.

As shown in Fig. 4, following IR, AICAR treatment markedly increased the expression of LC3-II (2.0±0.2) compared with that of IR-only-treated CNE-2 cells (1.0±0.1; P<0.05). This indicated that increased AMPK levels promoted the expression of LC3-II in CNE-2 cells following IR The relative density of LC3-II in *PARP-I*-silenced cells following IR (0.7±0.1) was significantly decreased compared with that of IR-only-treated CNE-2 cells (1.0±0.1; P<0.05). In addition, Compund C treatment following IR (0.6±0.2) markedly decreased LC3-II expression compared with that of the IR-only treatment group (1.0±0.1; P<0.05), suggesting that inhibition of AMPK reduced the expression of LC3-II following IR.

### Effect of PARP-1 and AMPK expression on the proliferation rate of CNE-2 cells

**Figure 5:**
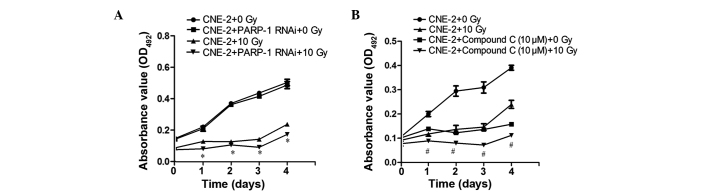
*PARP-1* or AMPK inhibition attenuates the proliferation of CNE-2 cells following 10 Gy IR, as determined using an MTT assay. (A) The *PARP-1* gene was silenced by RNAi using lentivirus-delivered small-hairpin RNA transfection. (B) Cells were treated with 10 *µ*M Compound C, an AMPK inhibitor, for 24 h. An MTT assay was then used to determine cell proliferation rates in the presence or absence of 10 Gy IR following incubation for 0, 1, 2, 3 and 4 days. Values are presented as the mean ± standard deviation. ^*^P<0.05, vs. CNE-2 and *PARP-1* RNAi + 0 Gy IR; ^#^P<0.05, vs. CNE-2 + Compound C (10 *µ*M) + 0 Gy IR. IR, ionizing radiation; PARP-1, poly-(adenosine diphosphate-ribose) polymerase-1; Compound C, 6-[4-(2-Piperidin-1-yl-ethoxy)-phenyl)]-3-pyridin-4-yl-pyrrazolo [1,5-a]-pyrimidine; AMPK, adenosine monophosphate-activated protein kinase; OD, optical density.

**Figure 6:**
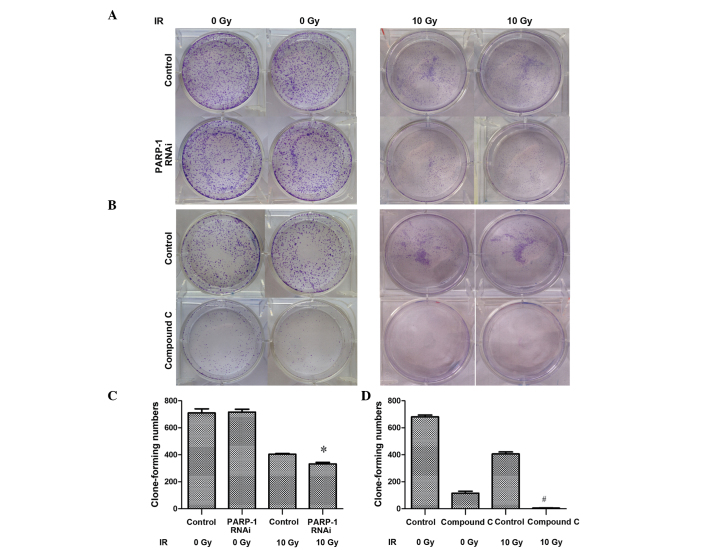
*PARP-1* or AMPK inhibition attenuates the proliferation of CNE-2 cells following 10 Gy IR, as determined using a clone formation assay. (A) The *PARP-1* gene was silenced by RNAi using lentivirus-delivered small-hairpin RNA transfection. (B) Cells were treated with 10 *µ*M Compound C, an AMPK inhibitor, for 24 h. A colony formation assay was used to determine cell proliferation rates in the presence or absence of 10 Gy IR, cells were seeded onto plates as a density of 5×10^3^. The number of colonies containing ≥50 cells were counted under a microscope to quantify the proliferation of (C) *PARP-1* RNAi-silenced cells and (D) Compound C-treated cells. Values are presented as the mean ± standard deviation. ^*^P<0.05, vs. CNE-2 + *PARP-1* RNAi with 0 Gy IR; ^#^P<0.05, vs. CNE-2 + Compound C (10 *µ*M) with 0 Gy IR. IR, ionizing radiation; PARP-1, poly-(adenosine diphosphate-ribose) polymerase-1; RNAi, RNA interference; Compound C, 6-[4-(2-Piperidin-1-yl-ethoxy)-phenyl)]-3-pyridin-4-yl-pyrrazolo [1,5-a]-pyrimidine; AMPK, adenosine monophosphate-activated protein kinase.

As shown Figs. 5 and 6, *PARP-1* gene silencing following IR significantly attenuated the proliferation of CNE-2 cells (P<0.05), demonstrating sensitization to IR. In addition, the AMPK inhibitor Compound C sensitized CNE-2 cells to IR, as indicated by the inhibition of proliferation (P<0.05). Furthermore in Compound C-treated cells without IR, CNE-2 cell proliferation was also significantly reduced compared with the control group (P<0.05).

## Discussion

Autophagy has been reported to be associated with cancer processes as well as protection against cellular stress. Autophagy, is a cellular degradation process which occurs at low basal levels in the majority of cell types for the maintenance of cellular homeostasis. It is important for the degradation and recycling of damaged proteins, organelles and other cytoplasmic constituents. Autophagy is induced following types of metabolic stress, including oxidative stress, nutrient starvation or endoplasmic reticulum stress, in order to supply nutrients and energy for promoting cell survival (34). Song *et al* (35) reported that autophagy acts as a protective mechanism response to the apoptosis induced by IR. In addition, it was suggested that tumors utilize autophagy as a survival mechanism to overcome the stresses imposed during cancer progression and those caused by radiation. However, when these stresses reach a critical point, autophagy was hypothesized to revert to mediating cell death (36). Several studies have indicated that pharmacologic or genetic inhibition of autophagy may enhance the effectiveness of cancer treatments by sensitizing cancer cells to radiation (22,37). The present study aimed to investigate the expression of upstream molecules of autophagy, including PARP-1, AMPK and mTOR in CNE-2 cells following IR. The results confirmed that PARP-1 regulated autophagy in CNE-2 cells following IR through activation of AMPK and the subsequent suppression of mTOR. In addition, it was demonstrated that Compound C inhibited the expression of p-LKB1-Ser428 following IR. Therefore, there are three key aspects of the present study which require discussion: The induction of autophagy through irradiation; the role of PARP-1, AMPK and mTOR as upstream molecules of autophagy; and the inhibition of p-LKB1-Ser428 expression by Compound C.

A previous study confirmed that 10 Gy IR induced autophagy and served as a cell survival mechanism against IR-induced cell death (22); therefore, 10 Gy IR was used in the present study. The results demonstrated that following IR the expression of LC3-I decreased in CNE-2 cells compared with that of the untreated control group; in addition, LC3-II levels were increased compared with those of the untreated control group. This indicated that LC3-I converted to LC3-II following IR, thus inducing autophagy.

Huang *et al* (25) identified the novel role of PARP-1 in autophagy, the mechanism of which proceeded via the LKB1/AMPK/mTOR pathway in order to enhance cell survival following H_2_O_2_ -induced oxidative stress in Bax^−/−^Bak^−/−^ MEFs. In the present study, CNE-2 cells were exposed to IR. p70S6 kinase is a downstream molecule of the mTOR pathway; therefore, the p70S6K expression may be used to indicate mTOR activity. In the present study western blot analysis was used to assess the protein expression of PARP-1, p-LKB1, p-AMPK and p-p70S6K following IR in different experimental groups, including cell transfected with LV-shRNA to silence *PARP-1* as well as treatment with the AMPK activator AICAR or the AMPK inhibitor Compound C. The results demonstrated that IR promoted the expression of PARP-1; however, AICAR and Compound C exhibited no significant effects on the expression of PARP-1. In addition, it was revealed that whether CNE-2 cells are treated with IR or not, they express p-LKB1-Ser428; notably, Compound C inhibited the expression of p-LKB1-Ser428. IR was demonstrated to promote the expression of AMPK in CNE-2 cells, which was further enhanced following AICAR treatment. By contrast, *PARP-1* gene silencing and Compound C treatment reduced the expression of AMPK following IR. Furthermore, IR reduced the expression of p-P70S6K in CNE-2 cells, which was further attenuated by AICAR. However, *PARP-1* gene silencing and Compound C induced the expression of p-P70S6K following IR. Subsequently, it was demonstrated that IR promoted the expression of LC3-II in CNE-2 cells, which was further enhanced following AICAR treatment. *PARP-1* gene silencing as well as Compound C treatment were found to reduce the expression of LC3-II following IR. Consequently, it was concluded that IR induced the activation of PARP-1, p-AMPK and LC3-II as well as inhibited the expression of p-P70S6K. Activation of AMPK had no impact on PARP-1 expression, whereas it was demonstrated to promote the expression of AMPK and LC3-II as well as inhibit p-P70S6K expression. By contrast, *PARP-1* gene silencing inhibited the expression of AMPK and LC3-II as well as activated the expression of p-P70S6K. AMPK inhibition reduced the expression of AMPK and LC3-II as well as activated p-P70S6K expression, while it exhibited no impact on PARP-1 expression. Overall, these results implied that PARP-1 promoted autophagy through the AMPK/mTOR pathway in CNE-2 cells following 10 Gy IR.

DNA damage has been shown to be generated by irradiation (38); such damage may lead to PARP-1 activation. The activation of PARP-1 initiates in the production of large quantities of PAR polymers and the subsequent translocation of apoptosis-inducing factors from the mitochondria to the nucleus, resulting in programmed necrotic cell death (39,40). In turn, the transient accumulation of PAR and its metabolism induces a reduction in ATP and NAD^+^ levels as well as an increase in cellular AMP. Furthermore, AMPK activation results in the inactivation of the mTOR complex 1 pathway via Raptor phosphorylation and P70S6K inhibition. Therefore, mTOR complex 1 inhibition contributes towards the establishment of autophagy (41).

Compound C, a cell-permeable pyrrazolopyrimidine compound, was reported to have an inhibitory effect on kinase insert domain receptor/vascular endothelial growth factor receptor 2, activin receptor-like kinase (ALK)-2/bone morphogenetic protein receptor-I and AMPK kinase activity, while further exhibiting an attenuating effect on ALK-5/transforming growth factor β receptor type 1, ZAPK, spleen tyrosine kinase, protein kinase Cθ (PKCθ), protein kinase A (PKA) and Janus kinase 3. The results of the present study demonstrated that Compound C blocked the expression of p-LKB1-Ser428. The are several potential reasons for this. LKB1 phosphorylation at Ser-428 by ribosomal protein S6 kinase A1 and/or PKA is required to inhibit cell growth (42) and since Compound C reduces PKA it may reduce or inhibit the expression of p-LKB1-Ser428 concomitantly. In addition, activation of PKC induces LKB1-S428 phosphorylation (43); therefore, the expression of p-LKB1-Ser428 may be due to the Compound C-induced inhibition of PKCθ. Furthermore, Compound C may promote enzyme activity to degrade p-LKB1. LKB1 is a known tumor suppressor gene, which is an upstream molecule of AMPK kinase involved in the regulation of energy metabolism (44). In the present study, limited expression of p-LKB1-Ser428 was observed in CNE-2 cells, which was too small to detect the changes in expression between groups. IR did not appear to influence the expression of p-LKB1-Ser428 and *PARP-1* silencing had no observable impact on LKB1-Ser428 expression. However, LKB1 is phosphorylated at numerous sites in human cells, including Thr-363, Ser-428 and Ser334; the present study only detected the site of Ser428, which may account for the limited expression of p-LKB1.

In the present study, MTT and clone formation assays demonstrated that PARP-1 or AMPK inhibition attenuated the proliferation of CNE-2 cells. This therefore indicated that inhibition of autophagy contributed to the radiation sensitization of CNE-2 cells. In addition, it was observed that CNE-2 cells treated with Compound C without IR exposure demonstrated reduced proliferation compared with that of the control group. This may be due to the fact that autophagy occurs at low basal levels in the majority of cell types in order to maintain cellular homeostasis (34). Without IR, CNE-2 cells retain low basal levels of autophagy and the artificial downregulation of autophagy contributes to cell death. A previous study demonstrated that Compound C had a slight, but significant, anti-proliferative and anti-migratory actions on endothelial cells (45).

In conclusion, the results of the present study revealed that PARP-1 promoted autophagy through the AMPK/mTOR pathway in CNE-2 cells following IR. In addition, it was determined that the inhibition of PARP-1 or AMPK contributed to the radiation sensitization of CNE-2 cells. LKB1 expression was limited and it was not possible to detect the changes in LKB1 protein levels in CNE-2 cells; therefore, the results did not determine that PARP-1 promoted autophagy through the LKB1/AMPK/mTOR pathway in CNE-2 cells following IR. However, using ELISA or mass spectrometry in combination with western blot analysis may solve this issue for future studies.
